# Spatio–Temporal Heterogeneity of Urban Expansion and Population Growth in China

**DOI:** 10.3390/ijerph182413031

**Published:** 2021-12-10

**Authors:** Shuangshuang Liu, Qipeng Liao, Yuan Liang, Zhifei Li, Chunbo Huang

**Affiliations:** 1 Research Center for Spatial Planning and Human-Environment System Simulation, School of Geography and Information Engineering, China University of Geosciences, Wuhan 430078, China; liushuangshuang@cug.edu.cn; 2 School of Arts and Communication, China University of Geosciences, Wuhan 430078, China; liaoqp@cug.edu.cn (Q.L.); liangyuan612@cug.edu.cn (Y.L.); 3 Yongyehang (Hubei) Land and Real Estate Appraisal Consulting Co., Ltd., Wuhan 430060, China; zhifeiliir@gmail.com

**Keywords:** urbanization, population growth, urban expansion, inharmonious development, decoupling analysis

## Abstract

Urbanization has become one of the hot issues of global sustainable development, and is mainly characterized by urban population growth and construction land expansion. However, the inharmonious development of urban expansion and population migration has brought serious challenges to urban planning and management. China is the largest developing country in the world, and the urbanization process has accelerated over the past decades. In this paper, decoupling analysis was used to demonstrate the spatio–temporal relationship between urban expansion and population growth in 321 prefecture–level cities in China, providing a reference basis for sustainable development. The results showed that China’s population, total GDP, and construction land area increased from 1990 to 2018. The rate of construction land expansion was larger in the eastern coastal and western regions than in the northeastern and central regions, but the population growth rate was not significantly different among these regions. According to the decoupling analysis, the relationships of population–GDP, construction land–GDP, and population–construction land were mainly weak decoupling, indicating that both the population growth and the construction land expansion lagged behind the economic development, and the population growth lagged behind construction land expansion. In addition, the results were analyzed based on China’s four economic regions. Population and construction land area changes in the northeastern provinces experienced a shift from weak decoupling to expansive negative decoupling, then presented a strong decoupling. The decoupling state of population–construction land in the west region was relatively stable. The relationship between population and construction land in the central regions was mainly weak decoupling, and some cities developed into strong decoupling. The relationship between population and construction land in the east region experienced a shift from strong decoupling to weak decoupling, then demonstrated expansive negative decoupling, mainly manifested in the Beijing–Tianjin–Hebei, Yangtze River Delta, and Pearl River Delta urban agglomerations. Therefore, the northeast region should take measures to promote regional population growth while reasonably controlling the expansion of construction land, the west region should focus on ecological protection and moderately attract population, the central region should control their population development and reasonably allocate land, and the east region should pay attention to and solve the citizenship problem of migrant workers in second–tier and third–tier cities when promoting new urbanization.

## 1. Introduction

With more than half of the global population now living in cities and towns, the world is witnessing unprecedented urban growth [1]. The latest Population Survey report released by the United Nations shows that urban land expansion is mainly caused by population growth. According to the predictive model, the global population would increase from 7.7 million in 2019 to 9.7 million in 2050, with an increase rate of 26% [2]. The consumption of land resources is positively related to urban population density. Urbanization has become one of the hot issues of global sustainable development concerns, which is mainly characterized by urban population growth and construction land expansion. Urbanization is a complex and dynamic process playing out over multiple scales of space and time [3,4]. The definition of urbanization has always been controversial, which varies across disciplines such as demography, geography, sociology, economics, urban planning, etc., [5]. However, its essential connotation is the natural historical process that the transformation of human production and lifestyle from rural to urban areas [6]. The quantitative research on urbanization development has mostly focused on its external manifestations, such as the number of towns, population and construction land area. At present, the international urbanization research mainly focuses on the following fields: the development of global cities and the spatial reconstruction of global urban systems in the context of globalization and informatization, the assessment system and dynamic monitoring of urbanization development levels, the typology of urbanization development and the choice of national (regional) model, the interaction between urbanization and resources and the environment, and the sustainable development of typical urban agglomerations. However, the practical orientation of domestic research is obvious, with strong policy guidance. The studies mainly include spatial patterns and regional models of urbanization, the relationship between urbanization and economic development, resource and environmental carrying capacity [7], and so on. China’s urbanization is described as unbalanced, and requires a trade–off between the spatial conversion of population and urban land expansion [1].

### 1.1. Population Urbanization

Population urbanization is defined as the process of population concentration in urban areas or population immigration from rural areas into urban areas [8]. The theory, driving mechanism, development level and direction, and impact effects of population urbanization have been studied since the early 1990s [9]. Most studies on China’s population urbanization were based on the urbanization rate of the resident population [10]. China’s urbanization entered a phase of rapid development since 1979, and the proportion of the urban population reached 51.27% in 2011, already exceeding the world average. According to the national seventh population census bulletin, the urbanization rate of China’s population reached 63.89% in the end of 2020, and the floating population consisted of 376 million people. Population migration is an important factor in generating regional economic equilibrium processes, while regional economic disequilibrium would drive population migration from rural areas into urban areas [11]. On the one hand, a population urbanization process would not only improve the income and consumption capability for the new urban residents, but also provide sufficient labor force for long–term growth and human capital accumulation. On the other hand, a booming urban economy pulls the rural labor work in cities by providing higher earnings.

### 1.2. Land Urbanization

Land urbanization was originally proposed in 2007, which was calculated by the proportion of a built–up area to the total area [12]. Land urbanization is a dynamic process, involving both the scale and rate of land types transformed into urban construction land within a certain period [13]. China has experienced rapid land urbanization since the mid–1990s. The land urbanization rate increased from 30.62% in 2000 to 54.77% in 2014, representing an average annual growth of 1.61%. As an important input factor and space carrier for urban economic development, land facilitates the construction of economic urbanization, which in turn promotes the realization of urban land values through continuous improvement of land markets [14]. In the process of socio–economic growth, urban development will continue to increase investment, introduce foreign investment and strengthen urban infrastructure construction. The increased intensity of population and economic activities will gradually generate more demand for construction land, thus promoting land urbanization [14]. Under the current urban–rural land system, financial system and performance evaluation mechanism, “land finance” and ‘‘land to attract investment’’ serve as the core strategies in delivering large amounts of ‘‘bonus’’ and promoting economic growth [15]. However, regional economic development and land urbanization have led to the expansion of construction land and destruction of the ecological environment [16]. Therefore, economic, land use, and ecological protection need to be weighed simultaneously in order to achieve sustainable urbanization.

### 1.3. Relationship between Population Growth and Urban Expansion

The labor force changes caused by population migration and the expansion of land for urban construction have generated economic and social differences between urban and rural areas, which have become an important driving force for urbanization. Land urbanization and population urbanization are closely linked: new construction land development may be demanded by various needs of the growing urban population [17], and the form and functions of construction land can affect the spatial distribution of the population [18]. However, existing studies [8,19,20] have proved that China’s population urbanization lags behind land urbanization which has brought some problems such as rural hollowing, the further increase of the urban–rural gap, the abandonment of arable land, etc. Meanwhile, the increasingly prominent human–land conflicts brought by rapid urbanization have been manifested in many aspects, such as increased soil erosion [21], decreased soil fertility [22], reduced cropland area [16], accelerated degradation of ecosystem functions [23], etc. The spatio–temporal relationship between population and construction land connects the social and physical aspects of urbanization, and determines how urbanization interacts with many environmental changes [24,25,26]. Therefore, studying the human–land coupled relationship of urbanization has positive implications for global environmental governance and sustainable development.

Several studies have focused on the relationship between population change and land expansion. For example, Mahmoud et al. [27] have analyzed the mechanisms driving urban human–land change. The quantitative relationship between population growth and land expansion has been assessed using a combination of measurement models and GIS spatial analysis methods [25,28]. In addition, domestic scholars have studied the spatial coherence and spatio–temporal coupling of population growth and construction land expansion [8,29,30]. These studies cover multiple spatial scales such as regions [31], provinces [32], cities [33,34], and urban agglomerations [35]. They also provide a wide range of content and comprehensive methods to analyze the spatio–temporal distribution, change characteristics and driving mechanism of human–land development from different perspectives [29]. At present, the main research methods of China’s population–construction land coupling relationship include the coordination degree model [8,30], the coupled development relationship index [36], and the decoupling analysis [32,34]. The coupling degree can not only reflect the interaction between the two factors, but also evaluate the overall quality of sustainable urbanization. However, it does not easily reflect the overall effectiveness and synergy between subsystems. The coordination degree mainly focuses on the benign interaction of system components in the development process [37]. Owning to the advantages that the results are not affected by the change of statistical dimensions and can provide additional details about the decoupling state [38], decoupling analysis is gradually being widely used in the study of relationships between two or more systems. The Tapio decoupling analysis was first introduced into the field of economics by Tapio, and it is used to study the decoupling of transportation volume increments and economic development [39]. Later, this method was gradually applied to studies on the environment and energy economy. In this paper, it was used to analyze the degree of decoupling between population urbanization and land urbanization. At present, most of the existing spatial and temporal population–construction land coupling studies in China are focused on the scale of individual cities (urban agglomerations) or provinces. Few studies focus on the decoupling relationship between the two. Moreover, the development of population urbanization and land urbanization has gone through a process from coupling to coordination, and then from decoupling to re-coupling. Therefore, the Tapio elastic decoupling analysis was used in this paper to analyze the temporal–spatial relationship between China’s population urbanization and land urbanization.

Here, we analyzed the relationships of population–economy, construction land–economy, and population–construction land in China by using the decoupling analysis method. The specific objectives are to (1) explore population urbanization and land urbanization from 1990 to 2018; (2) analyze the spatial and temporal relationships among population, construction land, and economy; and (3) demonstrate the spatio–temporal heterogeneity of construction land expansion and population growth. Our study could provide a reference basis for regional planning, formulating reasonable land policies and population control policies, promoting the human–land coordinated development, and realizing the sustainable development of urbanization.

## 2. Materials and Methods

### 2.1. Study Area

**Figure 1:**
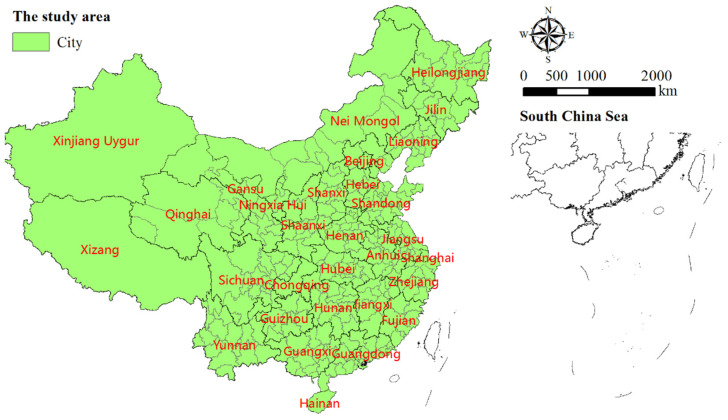
The study area.

The mainland of China is situated at 4° to 53°30′ North Latitude and 73°40′ to 135°05′ East Longitude, with an area of about 9.6 × 10^6^ km^2^. The topography is high in the west and low in the east, roughly in a stepped distribution. The west is mountainous and plateaus far away from the ocean, while the east is mostly plains and hills, close to the ocean. China is the most populous developing country in the world. According to the national seventh population census bulletin, its population has reached as high as 14.12 × 10^8^ by the end of 2020. In our study, 321 mainland Chinese administrative entities (cities) were selected, including 287 prefectural–level administrative regions, 30 province–administered counties, and 4 municipalities directly under central government control (Figure 1).

China’s urbanization has experienced rapid development over the past decades, but it still suffers from several problems such as insufficient resource and environmental carrying capacity, the imbalance between population urbanization, industrialization, and land urbanization. China’s demographic dividend is gradually disappearing after the urbanization process entered the stage of high–quality development, which means that cities will face huge demographic pressure in the future [40]. Tremendous demographic migration and socio-economic development have led to an unprecedented urban land expansion to accommodate urban dwellers and support the construction of urban infrastructure [41]. In addition, construction land increased exponentially by encroaching on vegetation types, which has become the largest driving factor for vegetation degradation [42]. Therefore, it is important to demonstrate the relationship between population and construction land, so as to coordinate the problems arising from the imbalanced development of urbanization and facilitate the high–quality and sustainable development of China’s new urbanization in the future.

### 2.2. Data Sources

The data of population, gross domestic product (GDP) and construction land area between 1990 and 2018 were collected. The population of urban and rural areas and GDP were derived from statistical yearbooks of the related provinces and municipalities in China. The construction land data were derived from land use/cover (LULC) maps which were obtained from the Resource and Environmental Science Data Center (http://www.resdc.cn (accessed on 12 September 2019)). Some prefectures were split up, merged, or abolished during 1990–2018, which resulted in a non–continuous statistic. Meanwhile, we also referred to China’s National Bureau of Statistics and local chronicles to complete the missing data.

### 2.3. Methods

The Tapio elastic decoupling analysis was used to investigate the relationships among population urbanization, land urbanization, and economic development. Tapio divided decoupling into 8 types: strong decoupling; weak decoupling; recessive decoupling; strong negative decoupling; expansive negative decoupling; weak negative decoupling; recessive coupling; and expansive coupling [39]. In this paper, a three–dimensional decoupling model was established, i.e., population–construction land decoupling, population–economy decoupling, and construction land–economy decoupling. The “elasticity decoupling model” of Tapio was adopted to document the decoupling relationship among them. The equation is as follows:

α=(Yt−Yo)Yo(Xt−Xo)Xo=ΔYΔX

where *α* is the decoupling elasticity coefficient; *t* and o refer to the number of years. In the dimension of population–construction land decoupling, *Y* and *X* are population and construction land respectively; Δ*Y* and Δ*X* denote the change rate of population and construction land respectively. In the dimensions of population–economy decoupling and construction land–economy decoupling, *Y* denotes population or construction land area; *X* denotes GDP change; Δ*Y* represents the change rate of population or construction land; and Δ*X* denotes the change rate of GDP.

**Figure 2:**
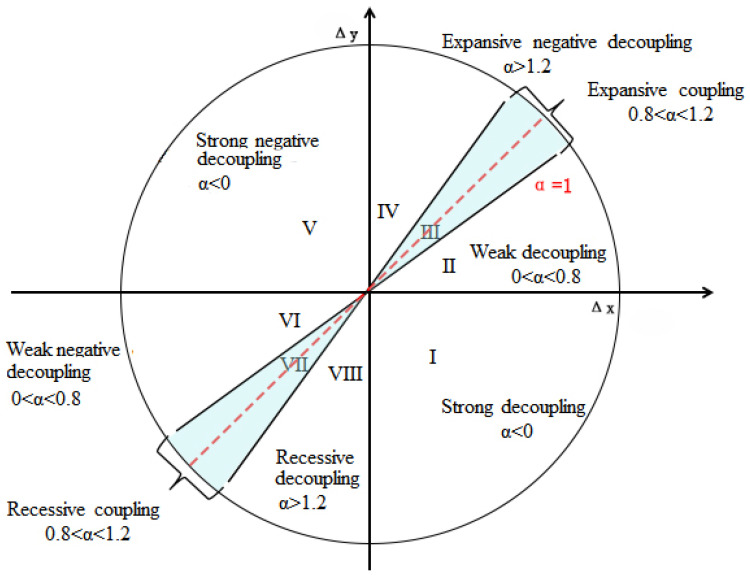
The degrees of coupling and decoupling of GDP, construction land expansion, and population change.

In this paper, the ±20% variation of elasticity values around 1.0 were still regarded as coupling to avoid overinterpreting slight changes as significant [39]. Based on the decoupling elasticity coefficients of a = 0.8 and a = 1.2, eight decoupling states were distinguished (Figure 2). The specific implications about their decoupling states are displayed in Table 1. Take the decoupling states of population and construction land, for example; strong decoupling occurs when construction land growth and population decline (elasticity coefficient is less than zero), indicating that development is extremely irrational. Weak decoupling, expansive negative decoupling and expansive coupling all occur when both construction land and population increase. Among them, the development under the weak decoupling state is not reasonable, which is characterized by the population growth rate is slower than that of construction land (elasticity coefficient is between 0 and 0.8). However, under the condition of expansive negative decoupling and expansive coupling, the development tends to be more reasonable.

## 3. Results

### 3.1. Dynamics of Population, GDP and Construction Land

#### 3.1.1. Spatio–Temporal Evolution of Population

The population increased significantly from 1029.49 million in 1990 to 1401.31 million in 2018, with a growth rate of 1310.85 (10,000 person)/yr, (Table 2). Population increased in most provinces, but the three northeast provinces (i.e., Heilongjiang, Jilin, and Liaoning) showed opposite trends around 2010, after which the population began to decline. The population of Zhejiang and Guizhou provinces decreased significantly during 2005–2010, and Hunan and Gansu provinces began to decrease after 2015. In addition, the population change rate varies among regions. Guangdong province had the highest population growth rate of 173.44 (10,000 person)/yr, followed by Guangxi, Hebei and Anhui provinces with relatively high rates of more than 100 (10,000 person)/yr over the past two decades. However, provinces like Qinghai, Jilin, Liaoning, Tianjin, and Tibet had lower population growth rates, where the values were below 10 (10,000 person)/yr. In particular, the population of Sichuan province showed a negative growth trend, with a change rate of −40.58 (10,000 person)/yr. However, its population reached a minimum at the end of 2010, before the population gradually began to pick up.

**Figure 3:**
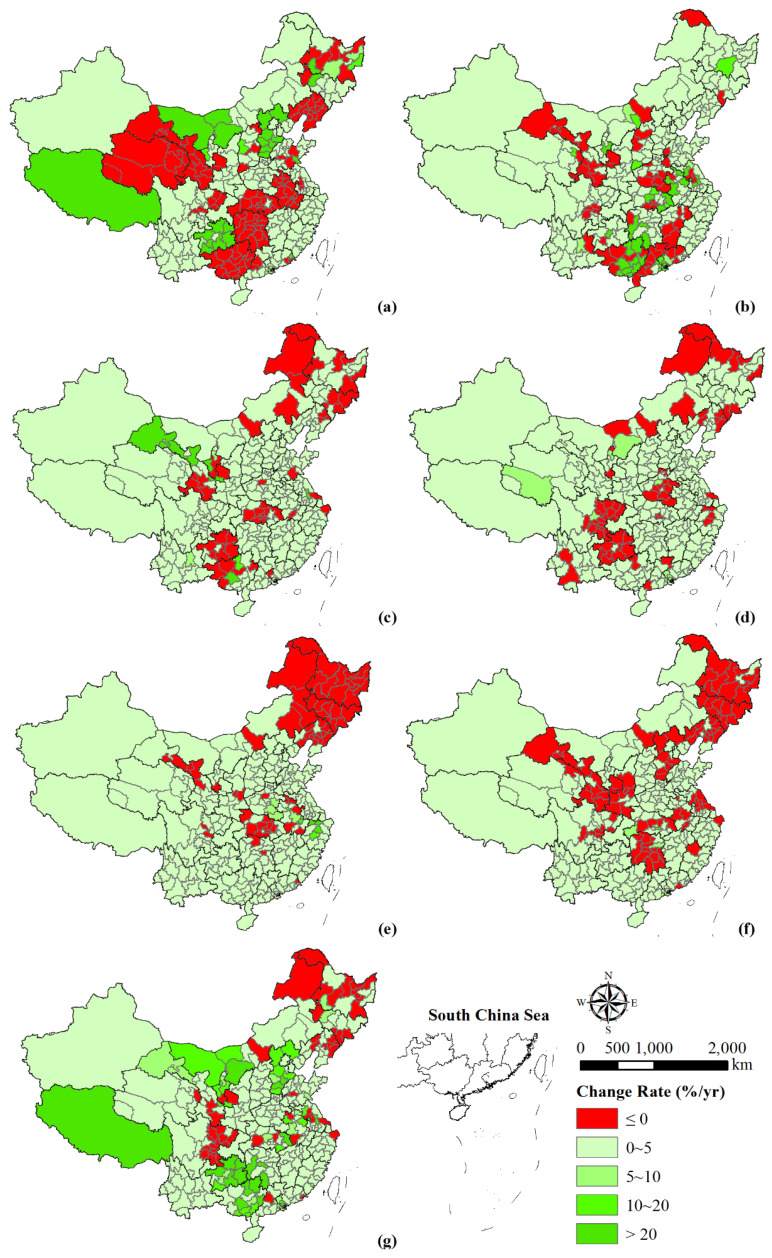
Spatial variation of the change rate of population during 1990–1995 (**a**), 1995–2000 (**b**), 2000–2005 (**c**), 2005–2010 (**d**), 2010–2015 (**e**), 2015–2018 (**f**), and 1990–2018 (**g**). Note: ≤0 denotes negative growth in population, 0–5 is low growth rate, 5–10 is medium growth rate, 10–20 is rapid growth rate, and >20 is high growth rate.

China’s population change was dominated by a low growth rate in the past few decades. The population change rates varied among different time intervals (Figure 3). During 1990–1995, the population of 119 cities showed negative growth, with 18 of them (mainly in Shanxi, Jilin, Heilongjiang, Jiangsu, Shandong, Guangdong and Sichuan provinces) decreasing significantly. Furthermore, 22 cities experienced a high growth rate in population, mainly in Hebei province, southwestern Inner Mongolia, Tibet, and Guizhou province. The remaining cities’ population presented a low growth rate. During 1995–2000, 58 cities experienced a negative population growth, mainly distributed in the southern parts of Henan and Jiangxi provinces, Gansu province, and the western parts of Guangxi and Guangdong provinces. In addition, cities with high growth rates were mainly located in the south of the country. During 2000–2005, there existed 49 cities with negative population growth, mainly in Guizhou province, eastern Hubei province, northwestern Guangxi province, southern Gansu province, and the northeast of the county. There were only seven cities with high population growth rate, four of which were in southeastern Gansu province. During 2005–2010, population decreased in 64 cities, mainly located in Guizhou, eastern Sichuan, southern Henan, and northwestern Heilongjiang provinces. Meanwhile, nine cities and regions, including Ordos, Zhengzhou, Guangzhou, Shenzhen, Dongguan, Zhongshan, Chengdu, Yinchuan, and Yushu Tibetan Autonomous Prefecture, showed medium growth rate. During 2010–2015, Jiaxing, Huzhou, Shaoxing, and Tongling showed a high population growth rate. During 2015–2018, population decreased in 95 cities which were distributed in the three northeastern provinces, as well as the provinces of Hebei, Hunan, Gansu, Shaanxi, Hubei, Jiangsu, and Sichuan. During this time Enshi, Tianmen, Changsha, and Zhuhai had a medium growth rate. In addition, Xiantao city showed the highest growth rate during this period, with an average annual growth rate between 10 and 20.

Overall, most cities showed an upward trend at a low population growth rate during 1990–2018 (Figure 3g). In particular, the urban population of individual regions grew rapidly, such as Guizhou, Tibet, Guangxi, Hebei, and Inner Mongolia. However, 50 cities experienced negative population growth, which were mainly distributed in the three northeastern provinces, eastern Sichuan, and southeastern Gansu provinces.

#### 3.1.2. Spatio–Temporal Evolution of GDP

According to the temporal dynamics of GDP (Table 3), China’s economic development exhibited an upward trend, with the total GDP expanded by 33,552.69 (100 million yuan)/yr from 1990 to 2018. The GDP changes varied among regions in different periods. Most provinces showed an exponential increase in GDP since 1990, while some provinces such as Shanxi, Inner Mongolia, and Liaoning displayed a decreasing trend after 2015. Furthermore, the change rate of GDP differed among regions from 1990 to 2018. Guangdong and Jiangsu provinces had the highest GDP change rates, which were 3498.55 (100 million yuan)/yr and 3266.79 (100 million yuan)/yr, respectively, followed by Shandong province. Tibet and Qinghai had the lowest GDP change rates, with values of 48.87 (100 million yuan)/yr and 91.32 (100 million yuan)/yr, respectively.

The output values of three industries in 31 provinces showed an increasing trend over time wholly. The primary industry output value in Shandong province was higher than that of other provinces, with a change rate of 147.67 (100 million yuan)/yr (Table S1). However, Henan province presented the highest change rate, increasing by 153.44 (100 million yuan)/yr. In comparison, Shanghai, Beijing, and Tibet had lower primary industry output value, with change rates of 2.92 (100 million yuan)/yr, 3.07 (100 million yuan)/yr, and 3.90 (100 million yuan)/yr, respectively.

According to the changes in the secondary industry output value (Table S2), Shanxi and Shandong ranked first in 1990, while were exceeded by Jiangsu and Guangdong in 1995. Their output value grew to 426.70 billion and 415.26 billion by 2018, at change rates of 1508.32 (100 million yuan)/yr and 1512.01 (100 million yuan)/yr, respectively, which were much higher than other provinces. In addition, Tibet, Hainan, Ningxia, and Xinjiang had lower output value, and their change rate was significantly lower than that of other provinces, with Tibet exhibiting the minimum change rate of 19.88 (100 million yuan)/yr.

The tertiary industry output value increased from 9.31 billion in 1990 to 488.61 billion in 2018 with a change rate of 16,767.48 (100 million yuan)/yr, which varies among provinces significantly (Table S3). The output value of Shandong province in 1990 was 147,499 million, far exceeding Guangdong province, which ranked second with a value of 49,243 million. Tibet exhibited the lowest tertiary industry output value, with only 1003 million. This gap increased further by 2018. The output value of Guangdong province was 5,568,920 million, which was several dozen times higher than that of Tibet. In terms of change rate, provinces with the largest change rate of tertiary industry output value in GDP were Guangdong and Jiangsu, with values of 1913.51 (100 million yuan)/yr and 1803.46 (100 million yuan)/yr, respectively, while Tibet was only 25.09 (100 million yuan)/yr.

#### 3.1.3. Spatio–Temporal Evolution of Construction Land Expansion

The construction land area increased by 1989.90 km^2^/yr, from 25,799.36 km^2^ in 1990 to 81,118.79 km^2^ in 2018 (Table 4). The change rate of construction land area varied among regions. Shandong province had the the highest change rate of 275.26 km^2^/yr, while the construction land area of Qinghai province only increased from 111.65 km^2^ in 1990 to 204.66 km^2^ in 2018 with a growth rate of 3.6 km^2^/yr.

**Figure 4:**
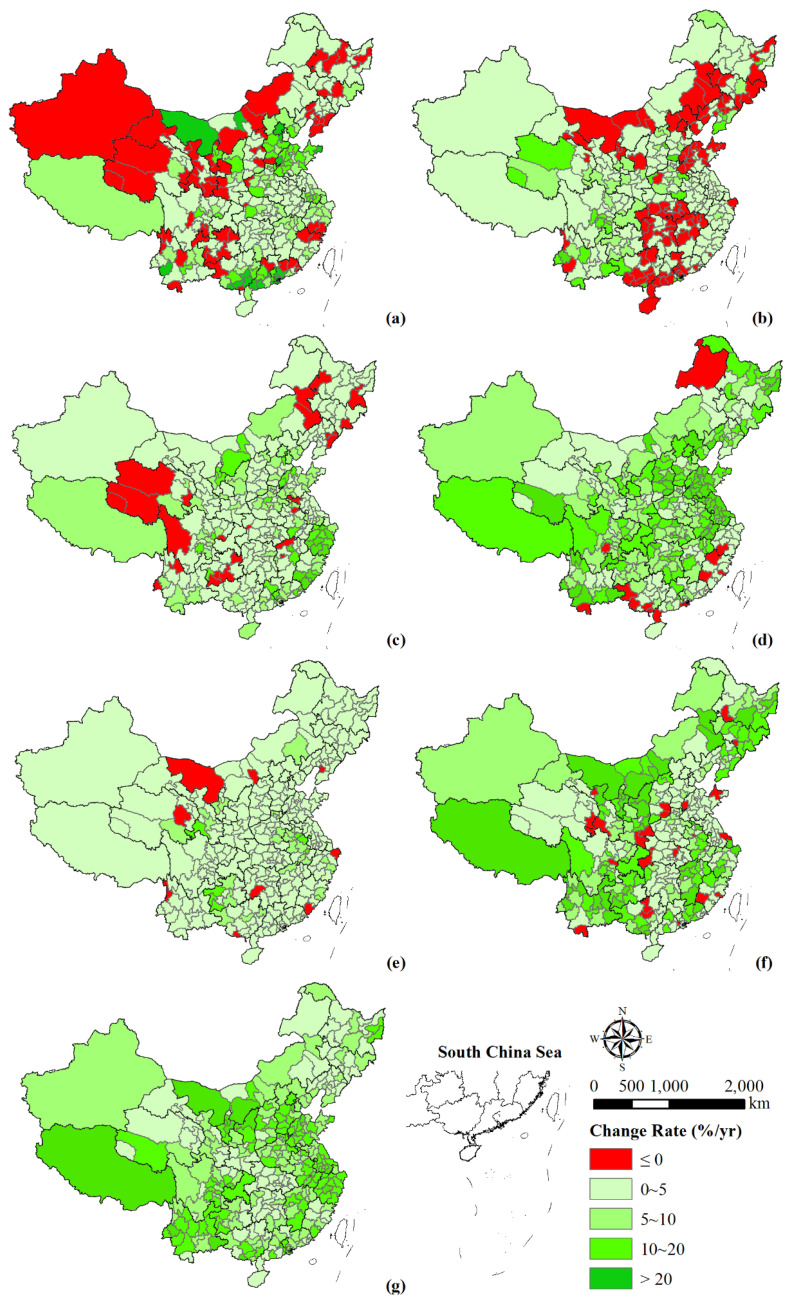
Spatial variation of the change rate of construction land during 1990–1995 (**a**), 1995–2000 (**b**), 2000–2005 (**c**), 2005–2010 (**d**), 2010–2015 (**e**), 2015–2018 (**f**), and 1990–2018 (**g**). Note: 0–5 is low–speed expansion, 5–10 is medium–speed expansion, 10–20 is rapid–speed expansion, and >20 is high–speed expansion.

The construction land expansion rate varied among different time intervals (Figure 4a–f). During 1990–1995, 19 cities had rapid–speed expansion, mainly located in Guangdong and Guangxi provinces. Eight cities and regions, including Beijing, Langfang, Weihai, Liaocheng, Jiyuan, Lincang and Inner Mongolia, presented high–speed expansion. However, 67 cities showed a state of negative growth, which was mainly distributed in the northeast, northwest, and southwest regions of China. From 1995 to 2000, most cities were in a state of low–speed expansion, and only Handan, Yangquan, Beihai, and Baoshan exhibited high–speed expansion. Furthermore, some cities mainly located in the central region (i.e., Hubei, Hunan, and Jiangxi) presented a decrease in construction land area. From 2000 to 2005, the expansion rate of construction land in eastern coastal cities increased significantly, and the negative growth of construction land in the central region improve. From 2005 to 2010, few cities exhibited low-speed expansion. In particular, Shandong and Jiangsu provinces showed a significant trend of high–speed expansion. The urban expansion rate in the southeast coastal provinces declined, and some cities even experienced negative growth, such as Lishui, Nanping, Zhanjiang, Beihai, etc. From 2010 to 2015, most cities presented low–speed expansion. Seven cities and regions including Wuhu, Bengbu, Chuzhou, Liupanshui, Southwestern Guizhou Buyi and Miao Autonomous Region, Bijie, and Gannan Tibetan Autonomous Region expanded rapidly in construction land. In addition, eight cities and regions experienced negative growth in construction land area. From 2015 to 2018, 37 cities across the country experienced a high–speed expansion. Among them, cities in the northwestern and southwestern regions of China such as Xi’an, Yinchuan, Guizhou, Kunming, Lijiang, etc., and others such as Hohhot, Erdos, Harbin, Shanghai, and Lishui changed significantly. In addition, the construction land area of 24 cities and autonomous prefectures from 15 provinces decreased, such as Liaoyuan, Daqing, Huainan, Yantai, Liuzhou, etc.

The construction land area of all 321 cities increased from 1990 to 2018 except for Tongling, and the construction land expansion rates varied among regions (Figure 4g). Overall, the growth rate of construction land in southern China was faster than that in the north, and the cities in eastern coastal and western regions were faster than that in northeastern and central regions, especially in the Beijing–Tianjin–Hebei, Yangtze River Delta, and Pearl River Delta agglomerations. In addition, the construction land area varied significantly among cities from the same province. For example, Yulin and Yan’an in the north of Shaanxi province expanded faster than cities in the south, and the construction land of Jixi and Shuangyashan in the three northeastern provinces expanded much faster than other cities. Moreover, Hefei, Wuhu, Anqing, Huangshan, Chuzhou, Suizhou, Xuancheng, and Chizhou in Anhui province expanded rapidly. Huainan expanded at a low rate, while Tongling experienced negative growth.

### 3.2. Population–Economy Decoupling

**Figure 5:**
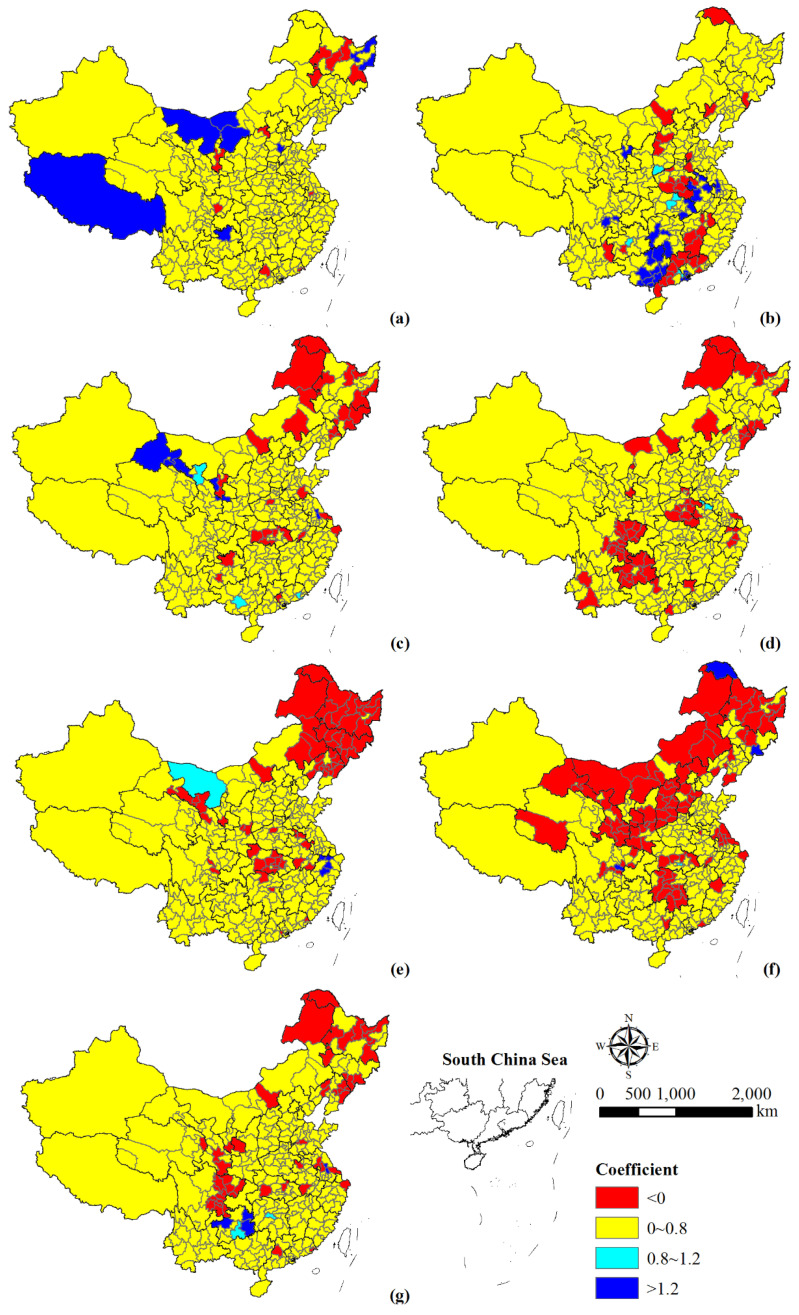
Spatial variation of the elasticity coefficient between population and GDP during 1990–1995 (**a**), 1995–2000 (**b**), 2000–2005 (**c**), 2005–2010 (**d**), 2010–2015 (**e**), 2015–2018 (**f**), and 1990–2018 (**g**).

China’s urban population and GDP exhibited four decoupling states, namely strong decoupling, weak decoupling, expansive negative decoupling, and expansive coupling (Figure 5). Four cities and regions were expansive negative decoupling, including Taizhou, Tongren, Bijie, and Qiandongnan Miao and Dong Autonomous Prefecture. This implied that the population growth rate was higher than that of GDP. The relationship in Loudi and Qiannan Buyi and Miao Autonomous Prefectures was expansive coupling, which was characterized by similar growth rates. Furthermore, 50 cities and regions showed strong decoupling with annual population change rate <0 and annual GDP change rate >0. They were mainly located in Heilongjiang, Jilin, southeastern Gansu, northeastern Liaoning, and eastern Sichuan provinces. The rest showed weak decoupling, with an elasticity coefficient between 0 and 0.8, indicating that the population growth rate was lower than that of GDP.

In addition, the elasticity coefficients between population and GDP varied across time (Figure 5a–f). During 1990–1995, eight cities and regions with expansive negative decoupling were mainly distributed in Heilongjiang province, Inner Mongolia and Tibet regions. Another 10 cities had a strong decoupling relationship between population and GDP. In addition, the population of Wuzhong and Guyuan showed a strong negative decoupling relationship with GDP, meaning that population growth was accompanied by a decrease in GDP. During 1995–2000, there existed 26 cities whose elasticity coefficients were more than 1.2. Yangzhou, Neijiang, and Leshan showed recessive decoupling, characterized by simultaneous declines in population and GDP, while the remaining cities showed expansive negative decoupling, mainly including Guangxi, Anhui and Guangdong, Jiangsu, and Hubei provinces. In addition, a few cities showed strong decoupling states, mainly in Guangdong, Henan, and Jiangxi provinces. Only Chaoyang presented strong negative decoupling. Moreover, six cities presented an expansive coupling relationship, including Yuncheng, Jingmen, Suizhou, Foshan, Zhongshan, and Guiyang. During 2000–2005, 31 cities and regions with a strong decoupling state were mainly distributed in the Inner Mongolia region, Heilongjiang, Jilin, and Hubei provinces. In addition, Taizhou, Zhangye, Pingliang, Jiuquan, and Zhongwei presented expansive negative decoupling. Jieyang, Nanning, and Wuwei showed a state of expansive coupling, indicating that the population and GDP development were relatively synchronized. During 2005–2010, the condition of elasticity coefficient < 0 was concentrated in provinces of Sichuan, Guizhou, Henan, Heilongjiang, Liaoning, etc., where the population and economy were in a strong decoupling state. However, the change direction of population and GDP in Fuyang was opposite to the above cities, which showed a strong negative decoupling state. In addition, Suzhou city in Anhui province showed an expansive coupling relationship. During 2010–2015, the decoupling state of four cities in Zhejiang province was expansive negative decoupling, including Jiaxing, Huzhou, Shaoxing, and Jinhua. Moreover, the population and economic development of the Alashan League region and Tongling city showed expansive coupling. In addition, 65 cities with strong decoupling relationships were mainly in provinces and regions of Gansu, Inner Mongolia, and Hubei. Only Zhongshan showed a strong negative decoupling state. During 2015–2018, 27 cities with increased population and decreased GDP showed a strong negative decoupling relationship. They were mainly located in Shanxi, Inner Mongolia, and Liaoning provinces. In contrast, 71 cities with elasticity coefficients also less than 0 showed strong decoupling, which were mainly concentrated in Hebei, Heilongjiang, Jiangsu, Hubei, Hunan, Sichuan, Shaanxi, and Gansu provinces. In addition, the population growth rate and GDP growth rate of Baishan, Daxinganling region, and Ziyang presented recessive decoupling. Only Xiantao city had an expansive coupling relationship.

### 3.3. Construction Land–Economy Decoupling

**Figure 6:**
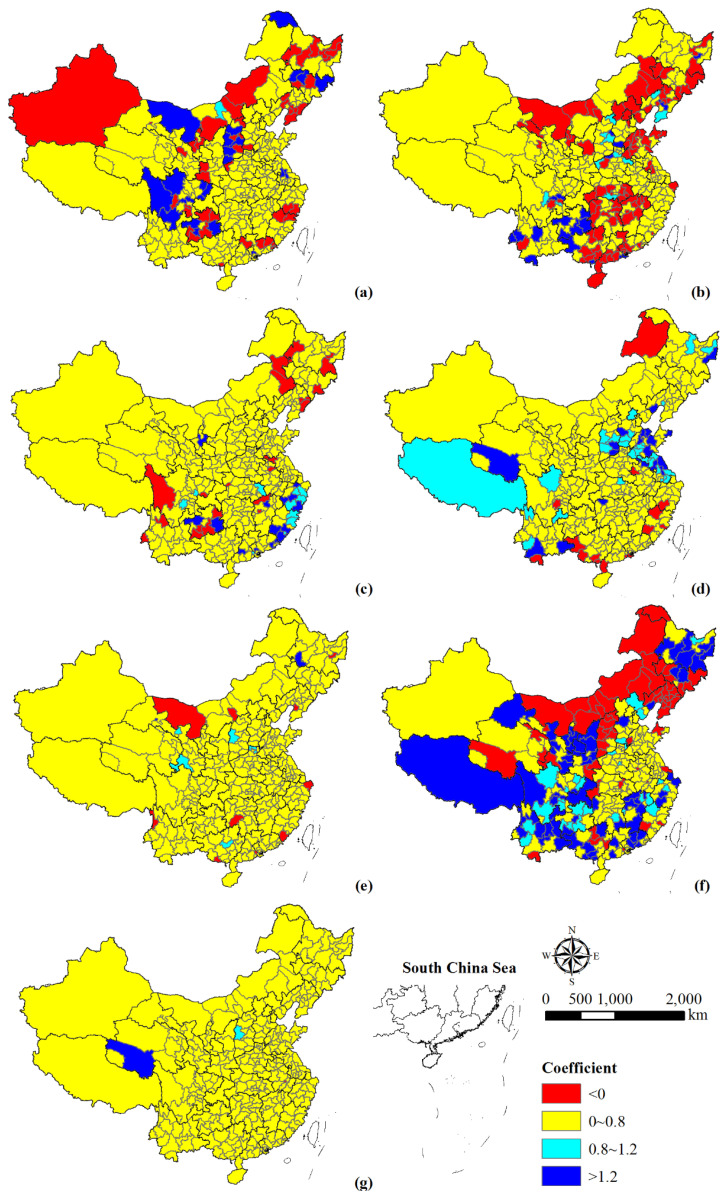
Spatial variation of the elasticity coefficient between construction land and GDP during 1990–1995 (**a**), 1995–2000 (**b**), 2000–2005 (**c**), 2005–2010 (**d**), 2010–2015 (**e**), 2015–2018 (**f**), and 1990–2018 (**g**).

The relationship between construction land and economy was weak decoupling as a whole (Figure 6). The total construction land area and GDP of all the 321 cities increased, with 319 cities having an elasticity coefficient between 0–0.8. This indicates that construction land grew at a slower rate than GDP. Moreover, the elasticity coefficient of Yushu Tibetan Autonomous Prefecture was >1.2, which presented an expansive negative decoupling state. This indicates that the growth rate of construction land was faster than that of GDP in this area. The elasticity coefficient of Lvliang was between 0.8 and 1.2, in which construction land and economic development were more synchronized and exhibited an expansive coupling relationship.

In addition, the decoupling state of cities varied across time (Figure 6a–f). From 1990 to 1995, 24 cities mainly in Shanxi, Sichuan, Jilin, and Guizhou provinces had an expansive negative decoupling relationship. Only Baotou showed expansive coupling. Furthermore, 48 cities with expanded construction land and decreased GDP showed a strong decoupling state. They were mainly distributed in Liaoning, Heilongjiang, Xinjiang, Inner Mongolia, Shaanxi, the border of Zhejiang and Fujian, Guangdong, and Guizhou provinces. However, the changes in construction land and GDP in Jiamusi and Wuzhong were the opposite, and they showed a strong negative decoupling relationship. In addition, Guyuan presented weak negative decoupling, indicating that its construction land and economy both decreased and GDP decreased faster. During 1995–2000, 19 cities with expansive negative decoupling were mainly distributed in Yunnan, Sichuan, and Guizhou regions. Another 10 cities with coordinated construction land and economic development demonstrated expansive coupling relationship, mainly located in Shanxi, Henan, and Sichuan provinces. Despite the elasticity coefficient in Jingzhou being between 0.8 and 1.2, its construction land and GDP growth rate were less than 0, showing a recession coupling state. Meanwhile, 90 cities nationwide indicated strong decoupling. Huai’an, Yangzhou, Neijiang, and Leshan, with decreased GDP and expanded construction land area, showed a strong negative decoupling state. In addition, cities including Chaoyang and Tianmen exhibited weak negative decoupling. During 2000–2005, 21 cities with a strong decoupling state were mainly distributed in the Inner Mongolia region, Yunnan, Sichuan, Guizhou, and northeastern provinces. Another 11 cities had an expansive coupling relationship, mainly located in Zhejiang and Fujian provinces. In addition, 15 cities presented a higher growth rate of construction land than GDP for the expansive negative decoupling relationship, which were mainly distributed in Zhejiang, Fujian, Guangdong, and Guizhou provinces. During 2005–2010, 13 cities showed strong decoupling, mainly in Guangxi, Guangdong, Fujian, and northeast Inner Mongolia. However, Fuyang was in a strong negative decoupling state. In addition, 19 cities in Shandong province and Jiangsu province showed expansive negative decoupling. Another 27 cities showed expansive coupling, mainly in Tibet, Shanxi and southern Hebei, Beijing, southwestern Shandong, and Jiangsu provinces. During 2010–2015, eight cities showed a strong decoupling relationship, and Qitaihe and Zhongshan showed a strong negative decoupling state. In addition, both of Daqing and Jiayuguan had growth and elasticity coefficients >1.2 between construction land and GDP, which showed expansive negative decoupling. Moreover, five cities and regions of Lvliang, Anyang, Laibin, Jinchang, and Gannan Tibetan Autonomous Prefecture exhibited expansive coupling. During 2015–2018, 72 cities exhibited an expansive negative decoupling relationship, mainly in the provinces and regions of Heilongjiang, Jiangxi, Guangdong, Guangxi, Sichuan, Chongqing, Yunnan, Tibet, Gansu, Ningxia, Shanghai, etc. Furthermore, a total of 19 cities exhibited strong decoupling, which were mainly distributed in the northwestern and southern regions of China. Meanwhile, 44 cities showed strong negative decoupling, which were mainly cities and regions in Shanxi province, Inner Mongolia, Jilin, Liaoning, and Daxinganling area of Heilongjiang province. Another 27 cities mainly distributed in the southwestern region had an expansive coupling state. In addition, Changye, Jincheng, Liaoyuan and Daqing showed weak negative decoupling, where the growth rates of construction land and GDP were less than 0, with a faster growth rate in GDP.

### 3.4. Population–Construction Land Decoupling

**Figure 7:**
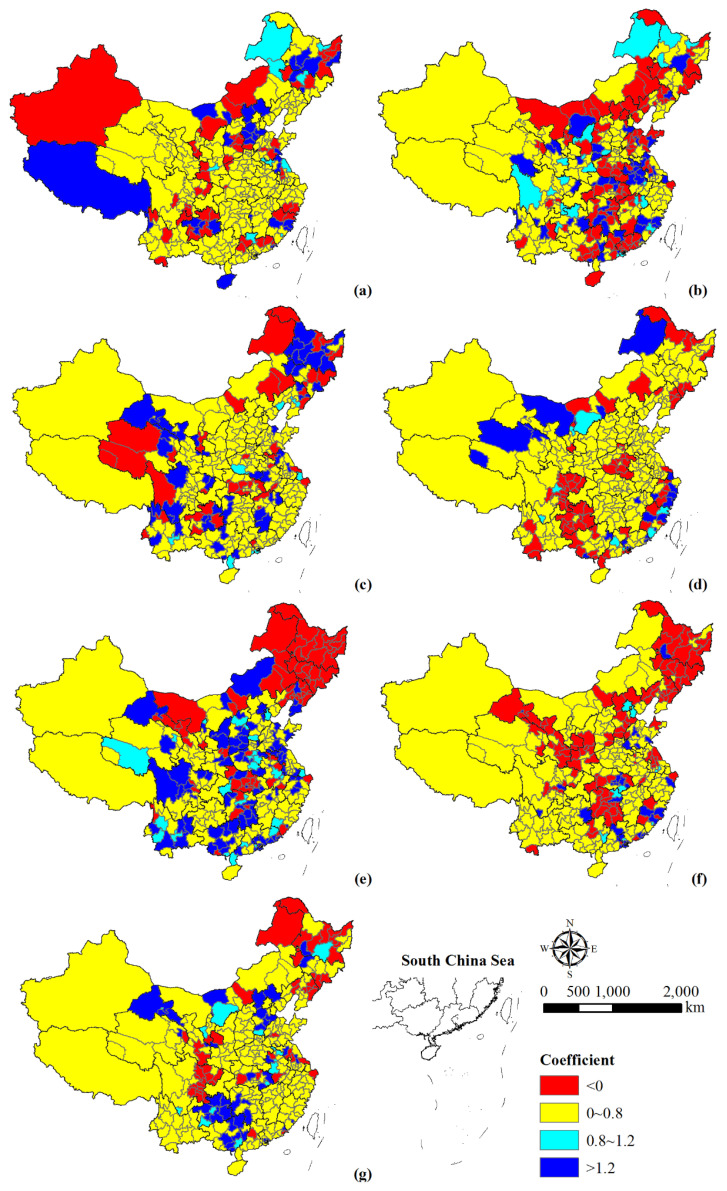
Spatial variation of the elasticity coefficient between population and construction land during 1990–1995 (**a**), 1995–2000 (**b**), 2000–2005 (**c**), 2005–2010 (**d**), 2010–2015 (**e**), 2015–2018 (**f**), and 1990–2018 (**g**).

The relationship between population growth and construction land expansion was weakly decoupled (Figure 7). There were 13 cities and regions in a state of expansive coupling, including Handan, Ordos, Harbin, Huainan, Suzhou, Lu’an, Huangshi, Yulin, Panzhihua, Guiyang, Qianxinan Buyi and Miao Autonomous Prefecture, Jiayuguan, and Zhongwei. In addition, cities with a negative decoupling relationship were mainly located in Guangxi, Guizhou, Gansu, and Hebei provinces. Cities of Heilongjiang, northeastern Liaoning, eastern Sichuan, and southeastern Gansu provinces presented a strong decoupling state.

In addition, the decoupling state of population growth and construction land expansion varied across regions at different times (Figure 7a–f). During 1990–1995, only nine cities and regions showed coupled population growth with land expansion, where the population growth rate and land expansion rate were >0. However, some cities in Tibet, Heilongjiang, Fujian, Guizhou, Hebei, and Hainan provinces presented negative decoupling. In addition, the cities with elasticity coefficients <0 showed strong decoupling or strong negative decoupling, which were mainly located in Xinjiang, Jiamusi, Mudanjiang, Zunyi, Lishui, Wenzhou, etc. During 1995–2000, cities with elasticity coefficients <0 were mainly located in the three northeastern provinces, Inner Mongolia, Shandong province, provinces of the central region (i.e., Henan, Hubei, Hunan and Jiangxi provinces), Guangdong province, and Guangxi province. Among them, some cities showed a strong negative decoupling state, such as Yizhou, Lvliang, Daxinganling, Huai’an, Yangzhou, Leshan, Anshun, and cities in southern Henan and northeastern Guangdong provinces. In addition, population growth and construction land expansion in some cities tended to be coupled, such as Hegang, Daqing, Chengdu, Luzhou, Liupanshui, and Tongchuan. During 2000–2005, seven cities showed an expansive coupling relationship. The cities with an expansive negative decoupling state were mainly distributed in Gansu, Heilongjiang, Jiangxi, and Sichuan provinces. The elasticity coefficients of some cities in Qinghai, Heilongjiang, Hubei, and Guizhou provinces were less than 0, which indicated a strong decoupling relationship. In addition, the growth rate of 10 cities’ construction land area was less than 0, which showed a strong negative decoupling state. During 2005–2010, most cities in Sichuan, Henan, and Guizhou provinces with decreased population and increased construction land area showed a strong decoupling relationship. In contrast, coastal cities in Zhejiang and Fujian provinces were mostly in an expansive negative decoupling state, while cities adjacent to them mostly exhibited strong decoupling. In addition, nine cities nationwide exhibited an expansive decoupling state. During 2010–2015, population and construction land in Hohhot, Alxa League, Zhoushan, Shaoyang, and Nujiang Lisu Autonomous Prefecture were strongly negative decoupled, while the three northeastern provinces, central Gansu province, central Hubei province, and eastern cities were mainly strongly decoupled. In addition, 25 cities and regions were in an expansive coupling state. During 2015–2018, the annual change rate of urban population in Northeast China, Hebei, Gansu, Shaanxi, Hubei, and Hunan provinces was <0 and the annual change rate of construction land was >0, showing a strong decoupling relationship. There were only eight cities where population and construction land were expansively coupled, namely Beijing, Tianjin, Wuxi, Xiamen, Xianning, Yueyang, Dongguan, and Wuzhou, indicating the two were more coordinated.

## 4. Discussion

### 4.1. Chinese Population, Economy and Construction Land Changes

#### 4.1.1. Population Growth in China

Population increased in most provinces (Table 2), but decreased in the three northeastern provinces (i.e., Heilongjiang, Jilin, and Liaoning). Their population loss is mainly the result of the lack of economic momentum caused by the transformation of resource–depleted cities [43]. In addition, the population change rate varied across regions significantly. Guangdong province had the largest population growth rate because of its high birth rate and the massive influx of external population. However, the provinces in western China had a lower population change rate, which might be related to their poor socioeconomic and natural environment. In particular, Sichuan province showed a trend of negative population growth. The reason may be that Chongqing established as a municipality directly under the central government attracts most of the population of Sichuan province. Its population gradually rebounded after 2010 with the development of the Chengdu–Chongqing urban agglomeration.

From a municipal perspective, cities with negative population growth increased, while cities that exhibited high growth rate decreased. Overall, the population change of Chinese prefecture–level cities tended to be balanced (Figure 3). Cities with a high population growth rate showed a tendency that shift from the western and northeastern regions to the central and southern regions from 1990 to 2000. The population change in the northeast region was mainly natural growth and intra–regional migration until 2000 [44]. However, the loss of labor force played a key role in the negative population growth in the following two decades. Meanwhile, the population of central and west regions (mainly in southern Henan, central Hubei, western Hunan, Gansu, Guizhou, and eastern Sichuan) presented a decreasing trend, which was mainly caused by the outflow of population. In addition, the population change of the eastern provinces presented significant spatial differences. Take Guangdong province, for example, the polarizing effect from the Pearl River Delta urban agglomeration produced population clustering. With the occurrence of trickle–down effect and the adjustment of relevant demographic policies, the population gradually tended to balance across the whole province [45]. In summary, although natural population growth resulted in regional population change, population movement plays a more important role in the total population change. The changing spatial pattern of population migration was driven by the social-economic dynamics and affected by the regulatory policies [46], thus causing the spatial differences of population urbanization.

#### 4.1.2. Chinese Economic Change

As the core indicator of national economic accounting, the exponential growth of the total GDP from 1990–2018 implied the rapid development of China’s economy (Table 3). Combining the contribution of the three industries’ output value to the total GDP, the development of the secondary and tertiary industries has a stronger driving effect on the overall economy than the primary industry. As one of the important components of the Yangtze River Delta and Pearl River Delta urban agglomerations, Jiangsu and Guangdong provinces concentrated a large number of industries and population with the location advantage after 1995 [47,48], accelerating the development of secondary and tertiary industries (Tables S2 and S3). Their total GDP in 2018 far exceeded other provinces. In addition, some other southeast coastal provinces, such as Zhejiang and Guangxi, also experienced significant economic growth. The central provinces (e.g., Shandong, Henan, Hubei, Hunan, etc.) have obvious advantages in land cost and sufficient labor force and resources compared to the western and eastern regions, and their primary industries are better developed [49]. However, large cities such as Beijing, Shanghai, and Tianjin had dense population and less land, where the primary industry output value was relatively low (Table S1). Moreover, the lack of impetus to the development of industrial structure caused by many factors (e.g., the sparse population, inadequate infrastructure, ecological fragility, and other factors) occurred in some western provinces, such as Qinghai, Tibet, and Ningxia. Their three industries developed more slowly, and the GDP change rate was far lower.

#### 4.1.3. Construction Land Expansion in China

The construction land areas showed continuous expansion since 1990, with the highest average annual growth rate in Shandong province and the lowest in Qinghai province (Table 4). From the perspective of prefecture–level cities, 320 cities exhibited different degrees of construction land expansion during 1990–2018 (Figure 4). Overall, the growth rate of construction land in southern cities was higher than that in the north, and cities in eastern coastal and west regions was higher than that in northeast and central regions. In addition, the construction land expansion rate varied among regions in different periods. A circular on arable land protection was issued by the General Office of the State Council of the People’s Republic of China in 1992, after which a series of arable land protection policies were established [50]. The construction land area of most cities was in a state of low–speed expansion or even negative growth from 1990 to 2005. However, the central government was expected to invest four trillion dollars to promote stable economic growth in response to the international financial crisis. Therefore, few cities exhibited a low-speed expansion on the rate of construction land during 2005–2010. Furthermore, adjustments in construction land management policies led to a low–speed expansion exhibited in more than 80% of the cities during 2010–2015. As several studies proved that the reduction of arable land mainly resulted from the occupation of construction land [42,51], land use policies should be better utilized as the main deterring forces to urban and infrastructure development.

### 4.2. Relationships among Population Expansion, Economic Development and Urban Expansion

#### 4.2.1. Relationship between Population Expansion and Economic Development

China’s population and GDP exhibited a weak decoupling relationship (Figure 5). The growth rate of population was lower than that of economy. Xiao et al. [52] pointed out that population changes were often triggered by economic development factors, thus exhibiting a certain lag, which was consistent with our study results. The northeastern China and Sichuan province showed strong decoupling. The push generated by the backward development of the local economy and the pull from the rapid development of neighboring areas contribute to their population loss. In addition, the decoupling relationship between population and GDP changes varied among regions in different development periods. For example, a strong decoupling state gradually shifted from the central and east regions (mainly the northwestern part of Guangdong province) of China to the northeast and west regions, which could be seen that the agglomeration of economic factors in China was higher than population, as evidenced by the study of Jiang et al. [53]. The emergence of the transfer direction illustrated the effectiveness that China’s regulatory policies have boosted the economy of the northeast and west regions.

#### 4.2.2. Relationship between Construction Land Expansion and Economic Development

Similarly, construction land and GDP exhibited a weak decoupling relationship from 1990 to 2018 (Figure 6), which indicated that the construction land expansion rate was lower than that of GDP. The area of construction land experienced significant changes at different times due to national policies, making it more volatile compared to the whole study period. The results indicated that the construction land expansion was lagging behind economic growth, which is consistent with the findings of Liu et al. [14] and Zhong et al. [54]. The decoupling relationship between construction land expansion and GDP change varied among regions in different periods. The northeast region mainly exhibited weak decoupling, then showed strong negative decoupling combined with expansive negative decoupling in some cities. However, the west region experienced the shift from weak decoupling to expansive negative decoupling combined with local expansive coupling. The reason may be the shift of economic gravity center from the northeast to the west under the development strategy [14]. Major initiatives include the Revitalization of Northeast China strategy, the Great Western Development strategy, the regional–integration strategy, the strategic concept of the 21st Century Maritime Silk Road, etc. In addition, the central region sustained a relatively stable decoupling relationship, which mainly showed weak decoupling. However, the decoupling state of Shanxi province changed significantly. Shanxi province mainly develops resource–dependent industries and lacks economic vitality in the context of industrial structure transformation. In addition, there was a trend shift from strong decoupling to a combination of expansive negative decoupling and expansive coupling, then demonstrated weak decoupling in the east region. As a pioneering area of national economic development, the corresponding market mechanism was being improved while the rapid economic development. These areas could better realize the substitution of technology and capital for land input, so that the dependence of economic development on land expansion gradually decreased [14].

#### 4.2.3. Spatio–Temporal Heterogeneity of Urban Expansion and Population Growth

From 1990 to 2018, China’s population growth and construction land expansion exhibited a weak decoupling relationship (Figure 7). The population growth rate was lower than that of construction land expansion, implying that the urbanization development of land and population was in an uncoordinated state. Moreover, the decoupling relationship between population growth and construction land expansion varied among regions at different development stages. The three northeastern provinces were mainly weak decoupling in the early development stages, then a state shift from expansive negative decoupling to strong decoupling occurred. This phenomenon is mainly caused by the population fluctuation, especially from 2010 to 2018 when the population showed negative growth. The northeast region should take measures to promote regional population growth under reasonable control of construction land expansion. Moreover, most cities in the west region showed weak decoupling and were relatively stable. Compared to other regions, the west region has better control over population and construction land. The decoupling relationship in the central region was mainly weak decoupling, and some cities developed into strong decoupling. There exists a relatively serious problem of extensive use of construction land and lower land intensive degree in the central region. Lagging-behind population urbanization itself reflects inadequate population conglomeration, ultimately resulting in a low land use efficiency [20].Therefore, the central region should pay attention to the intensive use of land resources in future planning and management. The decoupling performance of the east region was mainly in the Beijing–Tianjin–Hebei, Yangtze River Delta and Pearl River Delta urban agglomerations. They had gone through a shift from strong decoupling to weak decoupling, then experienced expansive negative decoupling, and some cities exhibited strong decoupling in recent years. This contrasts with the large number of migrant workers and temporary residence in the eastern urban areas. The east region should pay attention to and solve the problem of migrant workers in second–tier and third–tier cities in the process of promoting new urbanization [55].

### 4.3. Land Use and Landscape Planning Implications

**Figure 8:**
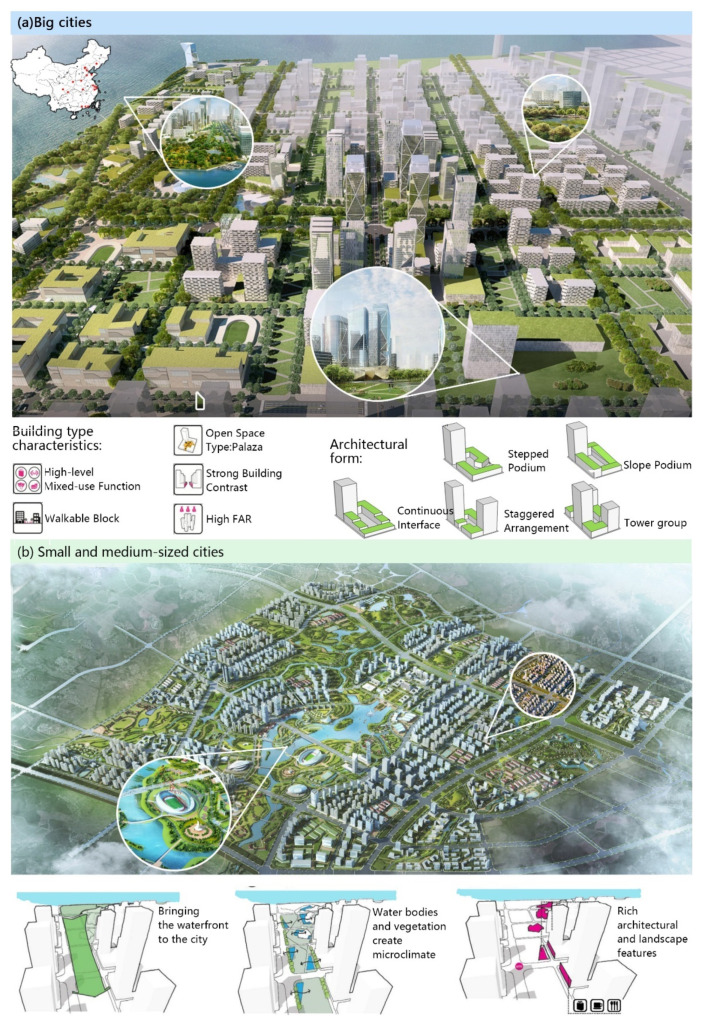
Land use and landscape planning implications for (**a**) big cities and (**b**) small and medium–sized cities.

It is a common phenomenon of urbanization process that the growth rate of urban land is larger than the growth rate of population. In this paper, we discussed the differences of the growth rates of construction land and population among cites. We divided Chinese cites to two types, i.e., big cities and small and medium–sized cities, to put forward targeted and creative planning and design solutions (Figure 8). In the study, big cities include Beijing, Shanghai, Guangzhou, Shenzhen, Chongqing, Tianjin, Wuhan, Chengdu, Dongguan, Nanjing, Hangzhou, Zhengzhou, Xi’an, Shenyang, Jinan, Qingdao, and Suzhou, which have an urban resident population of five million or more. Other cities with a population of less than five million are classified as small and medium–sized cities.

Due to the rapid social and economic development of big cities (Figure 8a), new urban construction land is growing rapidly. Urban development of big cities has increased urban constuction land density, leading to a constant compression of urban landscape space and a reduction in public open space. Therefore, it is recommended to strengthen the landscape use of the corner space in big cities and to innovate the type and form of architecture, so that the city can gain more open space, improve the quality of the urban landscape, improve the efficiency of land use, and reduce the damage to the landscape environment while ensuring economic development. Corner spaces, which exist either between buildings, at the bottom of tall buildings or around roads, have become a special spatial form in big cities. By developing mixed-use functional high-rise buildings, walkable block, open space type and other building types, and improving the level of intensive land use, corner spaces can be made into a characteristic spatial form in order to solve the situation of high density and low landscape faced by the development of big cities now.

As for small and mesium-sized cities (Figure 8b), the growth of urban land is faster than the growth of urban population. A large amount of farmland and water areas have been converted into land for construction, which has greatly damaged the landscape ecology and caused an increase in the fragmentation of the landscape. Therefore, it is recommended to pay attention to the original water systems and vegetation in the urban landscape planning and design stage so that they can be protected to enrich the diversity of species and maintain the balance of self-cycling ecosystems in the construction of natural ecology, as well as to enrich architectural and landscape features. At the same time, an urban expansion model that protects ecological safety should be adopted, controlling the rate of expansion of building land and ensuring the proportion of arable land, forest land, and other greenery to improve the ecological safety of the city. It should prevent and curb the disorderly expansion of urban land and improve the level of intensive land use; formulate and implement differentiated measures to regulate urban construction land and guide the rational use of land; strengthen the coordinated development of the relationship between urban people and the land; and construct reasonable urban land use.

### 4.4. Deficiencies and Future Research

The decoupling of population growth and construction land expansion involves social, political, and economic factors, and the dynamic linkages among regions will change this relationship as well. Our study purpose is to provide a scientific basis for the rational promotion of the national urbanization process. However, this study gives less consideration to the linkages and interactions between regions and lacks in–depth analysis of the driving factors that produce the above decoupling relationships. Therefore, the following aspects can still be made in future works based on existing study: (1) We will consider the interaction and spatial connection between regions, and attempt to construct a spatial econometric model to evaluate the interaction of human–land urbanization more objectively. (2) The study on the driving mechanism of the decoupling of human–land relationship will also be the key words of further in-depth research to explore and find the focus point of promoting the harmonious development of urban and rural human–land relationship. (3) The spatial agglomeration effects of urban construction land expansion and population growth and their decoupling relationships are combined to study, so as to better analyze the geospatial performance characteristics of the development of land urbanization and population urbanization in China.

## 5. Conclusions

China’s technological level and economic strength have continued to improve since the reform and opening up, and its urbanization process has undergone rapid development in the past decades. Meanwhile, it has also brought a series of problems, such as the idleness and waste of rural farmland, expanding urban–rural gap, urban environmental pollution, housing tension, etc. As two manifestations of urbanization, the harmonious relationship between population growth and land expansion is crucial to the healthy and sustainable development of urbanization. However, most of the current studies started from one aspect or on a small spatial scale, and few studies that analyzed the relationship between them from a national perspective. We selected 321 cities in mainland China and investigated the spatial and temporal heterogeneity of construction land expansion and population growth from 1990 to 2018 using the decoupling analysis method. Our results showed that the total population increased by 371.82 million, and most provinces presented an increasing trend over time. Furthermore, cities with negative population growth increased, while cities showing high growth rate decreased. The population change of Chinese prefecture–level cities tended to be balanced. Moreover, the output value of all three industries increased, and the development of the secondary and tertiary industries had a stronger driving effect on economy than primary industry. In addition, the total area of construction land showed exponential increase, and construction land expansion degrees varied among cities during different development periods. Overall, the construction land area in southern China grew faster than that in the north, and the eastern coastal cities and the west was faster than that in the northeast and central regions. On the other hand, by analyzing the decoupling between population growth, GDP change, and construction land expansion, we speculated on the possible reasons from the perspective of China’s four economic regions. The results showed that population growth and GDP, and construction land area and GDP, showed weak decoupling, implying that population growth and construction land expansion lagged behind economy development. The decoupling analysis of population and construction land area showed that population growth and construction land expansion exhibited an overall weak decoupling relationship, indicating that population urbanization and land urbanization in China were uncoordinated. The three northeastern provinces underwent a shift from weak decoupling to expansive negative decoupling, then exhibited a strong decoupling of population and construction land expansion. Their population fluctuated greatly, and measures should be taken to promote regional population growth under the condition of reasonable control of construction land expansion. The decoupling state of the west region was relatively stable. When implementing strategies such as the Great Western Development and the 21st Century Maritime Silk Road, ecological protection and attracting population should be focused on appropriately. The decoupling relationship in the central region was mainly weak decoupling, and some cities developed into strong decoupling. In the process of taking over the industrial transfer from the east region, the central region should control its population development and rational land allocation. The decoupling in the east region was mainly in the Beijing–Tianjin–Hebei, Yangtze River Delta and Pearl River Delta urban agglomerations. They experienced a shift from strong decoupling of population growth and construction land expansion to weak decoupling, then indicated expansive negative decoupling. The east region should pay attention to and solve the citizenship problem of migrant workers in the second–tier and third–tier cities when promoting new urbanization. In the context of sustainable urbanization in the new era, our research can provide a scientific basis for the coordinated development of human–land relationships.

## Figures and Tables

**Table 1:** Decoupling states and specific implications of the relationship among economy, construction land expansion and population change.

Decoupling States	Δ*X*	Δ*Y*	α	Specific Implication
Strong decoupling	>0	<0	<0	Extremely irrational development with decreasing *Y* and increasing *X*.
Weak decoupling	>0	>0	0–0.8	Both trends are increasing, but the increase in *Y* is less than that in *X*, making development less rational.
Expansive coupling	>0	>0	0.8–1.2	Both are on the increase and growing at a similar rate, with a reasonable development.
Expansive negative decoupling	>0	>0	>1.2	Both trends are increasing, but the increase in *Y* is more than that in *X*, making development to be rational.
Strong negative decoupling	<0	>0	<0	Increase in *Y* and decrease in *X*, intensification of *X* and rationalization of development when Δ*Y* is not below the minimum standard per capita.
Weak negative decoupling	<0	<0	0–0.8	Both *Y* and *X* are decreasing, and *Y* is decreasing at a slower rate than *X*, and development is becoming more rational.
Recessive coupling	<0	<0	0.8–1.2	Both are on a decreasing trend at a similar rate, with development tending to be reasonable.
Recessive decoupling	<0	<0	>1.2	Both trends are decreasing, but the decrease in *Y* is more than that in *X*, making development less rational.

**Table 2:** The temporal dynamics of population in China.

Total Population (10,000)								Change Rate (10,000 Person)/yr
Province	1990	1995	2000	2005	2010	2015	2018	
Beijing	1032.20	1070.30	1107.50	1180.70	1257.80	1345.20	1375.80	12.89
Tianjin	866.25	894.67	912.00	939.31	984.85	1026.90	1081.63	7.26
Hebei	2333.11	6420.47	6670.93	6864.65	7298.04	7650.83	7247.95	135.80
Shanxi	2897.23	3025.66	3247.80	3355.21	3574.11	3664.12	3718.34	30.47
Inner Mongolia	1931.33	2237.21	2375.54	2386.40	2472.19	2511.04	2533.98	18.48
Liaoning	4034.10	4034.10	4135.30	4189.10	4251.70	4229.70	4191.90	7.50
Jilin	2440.17	2550.87	2627.26	2669.37	2723.81	2662.08	2608.94	6.42
Heilongjiang	3120.00	3210.50	3698.00	3768.22	3842.79	3674.60	3574.40	18.87
Shanghai	1334.00	1414.00	1608.60	1890.26	2302.66	2415.27	2423.78	44.77
Jiangsu	6671.74	6520.14	6692.39	7252.88	7466.60	7717.59	7831.85	49.60
Zhejiang	4234.91	4369.63	4501.22	4602.10	3999.31	4873.34	4999.84	20.24
Anhui	4393.16	4393.16	5829.40	6062.37	6366.05	6949.11	7023.29	101.59
Fujian	2997.48	3136.77	3304.63	3384.95	3529.69	3720.68	3861.31	29.59
Jiangxi	3771.03	4062.54	4039.80	4311.24	4456.77	4565.63	4647.57	30.30
Shandong	8516.13	8701.16	8997.31	9248.18	9587.87	9847.16	10,047.24	55.70
Henan	8616.51	9183.00	9123.00	9771.79	9405.47	10,723.00	10,906.37	75.69
Hubei	4419.85	4465.69	5877.70	5836.52	6007.98	5989.41	6153.20	63.18
Hunan	5088.72	5088.72	6525.77	6732.10	6919.29	7240.84	6930.92	77.68
Guangdong	6959.89	6788.74	8525.19	9193.99	10,440.94	10,849.01	11,346.00	173.44
Guangxi	2026.30	2026.30	4210.46	4894.18	5159.47	5518.25	5659.17	141.84
Hainan	651.23	702.42	760.94	819.03	896.09	907.67	925.10	10.26
Chongqing	2920.90	3001.77	3091.09	3169.16	3303.45	3371.84	3403.64	17.96
Sichuan	9468.70	8989.50	8407.50	8642.10	8041.80	8204.20	8341.00	−40.58
Guizhou	635.55	3419.54	3755.72	3730.00	3478.94	3529.50	3600.00	67.31
Yunnan	3665.90	3844.78	4076.70	4450.60	4601.70	4741.80	4829.40	43.55
Tibet	35.68	239.84	259.83	280.31	300.22	323.97	343.82	8.53
Shaanxi	3275.02	3431.93	3572.17	3689.54	3854.84	3922.12	4003.99	25.80
Gansu	2076.07	2076.07	2143.10	2607.44	2689.89	2725.97	2556.62	25.10
Qinghai	456.25	456.25	480.42	503.91	549.97	573.94	586.77	5.19
Ningxia	351.33	396.66	587.86	596.20	632.96	667.88	688.11	12.02
Xinjiang	1529.16	1661.35	1849.41	2010.35	2181.58	2359.73	2486.76	34.28
Total	102,948.89	112,013.24	123,194.53	129,232.66	132,779.83	138,703.88	140,130.50	1310.85

**Table 3:** The temporal dynamics of GDP in China.

GDP (100 Million Yuan)								Change Rate (100 Million Yuan)/yr
Province	1990	1995	2000	2005	2010	2015	2018	
Beijing	500.80	1507.70	3212.80	7141.40	14,441.60	23,685.70	30,320.00	1068.74
Tianjin	310.95	931.97	1701.88	3947.94	9343.77	16,794.67	18,809.64	701.72
Hebei	469.76	2813.95	5354.18	10,263.55	20,616.05	30,441.36	34,896.90	1288.65
Shanxi	1494.94	3113.46	4450.44	10,678.23	24,234.59	34,163.83	16,759.99	982.23
Inner Mongolia	252.79	769.23	1417.71	4115.11	12,886.23	20,559.32	16,881.55	757.14
Liaoning	966.26	2997.35	4646.28	8765.61	20,650.82	28,674.56	25,732.80	1061.87
Jilin	1013.99	1941.53	3291.72	6720.65	16,328.70	26,418.63	27,789.35	1056.63
Heilongjiang	565.45	1603.43	3503.27	5394.79	11,391.05	15,523.30	16,606.50	624.21
Shanghai	781.66	2518.08	4812.15	9365.54	17,436.85	25,659.18	32,679.87	1141.22
Jiangsu	2383.06	5740.72	8168.67	18,661.77	41,849.64	73,071.81	94,323.54	3266.79
Zhejiang	838.24	3766.10	6719.29	13,470.26	27,033.92	43,038.40	56,425.57	1955.90
Anhui	566.35	1697.56	2626.97	5086.36	5086.36	12,173.44	30,429.42	809.13
Fujian	398.98	2124.27	4149.20	6567.71	14,462.75	25,923.10	35,788.57	1203.51
Jiangxi	401.99	1161.26	1868.07	3984.24	9431.45	16,852.56	22,003.32	762.57
Shandong	5107.24	5107.24	8582.50	19,115.88	40,184.22	63,072.52	77,871.60	2697.99
Henan	974.18	3085.92	5086.85	10,623.56	23,240.88	37,253.22	48,367.60	1683.73
Hubei	896.17	2804.22	4265.99	6561.03	15,790.42	31,058.53	40,860.09	1378.73
Hunan	670.15	2175.49	3645.83	6604.81	16,355.70	30,426.93	37,623.75	1325.68
Guangdong	1478.98	5891.03	10,596.65	22,972.59	47,713.28	75,740.92	101,025.94	3498.55
Guangxi	605.81	1164.69	1932.29	3992.55	9563.00	16,805.74	20,378.04	724.16
Hainan	102.42	363.25	526.82	918.75	2064.50	3702.76	4832.05	164.65
Chongqing	327.75	1123.06	1791.00	3486.22	7957.49	15,789.80	20,363.19	702.22
Sichuan	1252.69	2755.91	4139.92	7510.65	17,443.10	32,044.85	41,904.14	1423.55
Guizhou	824.16	945.41	1374.20	2038.44	4620.21	11,634.34	15,666.82	505.15
Yunnan	647.93	1226.91	2008.52	3492.63	7403.45	13,865.58	17,761.77	602.72
Tibet	27.70	56.11	117.80	248.80	507.46	1027.43	1477.63	48.87
Shaanxi	302.70	845.57	1762.01	3881.14	10,070.36	17,602.65	23,925.09	827.26
Gansu	445.02	598.86	962.47	1937.32	4042.15	6748.24	8184.19	284.66
Qinghai	323.41	353.02	394.56	540.11	1387.47	2359.89	2788.24	91.32
Ningxia	58.80	89.14	149.85	381.51	935.67	2916.27	3748.12	128.08
Xinjiang	274.01	814.85	1363.56	2604.14	5397.27	9235.57	12,199.08	416.46
Total	27,500.65	64,841.02	107,869.19	215,433.63	466,770.21	774,488.24	951,163.81	33,552.69

**Table 4:** The temporal dynamics of construction land in China.

Construction Land Area (km^2^)								Change Rate (km^2^/yr)
Province	1990	1995	2000	2005	2010	2015	2018	
Beijing	484.88	1081.42	1035.05	1299.42	2575.33	2629.70	2684.08	84.19
Tianjin	485.25	553.19	575.80	831.25	1167.16	1227.81	1288.45	32.52
Hebei	1161.04	1791.40	1927.76	2373.06	3927.92	4335.51	4720.12	132.37
Shanxi	771.51	771.37	986.02	1119.76	1916.49	1961.90	2307.29	58.73
Inner Mongolia	1109.08	1269.84	1161.43	1306.60	1567.78	1684.25	2791.18	44.97
Liaoning	1399.52	1371.01	1574.65	1643.42	2337.07	2394.45	3101.88	58.01
Jilin	907.39	940.99	1019.78	1095.70	1358.42	1477.04	1948.55	33.06
Heilongjiang	1269.32	1266.45	1366.92	1382.56	2092.88	2161.49	2839.73	52.83
Shanghai	562.72	761.23	778.54	916.03	1031.83	1079.19	1993.45	37.23
Jiangsu	1981.95	2618.90	2833.05	3369.52	7598.80	7973.37	8497.30	261.21
Zhejiang	810.52	1037.93	1145.87	2401.32	2535.20	3007.57	3603.40	101.77
Anhui	861.09	945.24	1015.99	1218.69	1903.36	2547.28	3167.47	80.89
Fujian	563.73	632.18	640.17	1238.79	1269.94	1430.46	1498.08	37.66
Jiangxi	468.89	599.41	584.81	849.61	1129.20	1220.43	1829.82	42.89
Shandong	2276.54	3588.07	3135.50	4057.02	8398.11	8940.91	9237.70	275.26
Henan	1323.43	1733.46	2161.83	2768.59	3731.12	4196.06	4780.10	125.38
Hubei	985.30	1220.72	1158.63	1311.32	1909.91	1950.11	2014.00	39.58
Hunan	838.76	961.02	956.51	1133.87	1701.77	1739.85	1904.60	40.92
Guangdong	1524.63	2968.36	2457.26	4206.77	5243.38	5380.66	5800.22	153.04
Guangxi	543.59	829.19	825.17	925.83	1003.75	1094.97	1356.45	23.17
Hainan	173.06	176.22	173.05	223.47	254.39	317.09	337.99	6.35
Chongqing	211.72	238.33	328.45	392.46	697.12	733.02	990.22	27.22
Sichuan	604.23	779.81	904.77	1187.26	1609.79	1671.20	2024.47	50.05
Guizhou	216.17	221.10	251.74	262.78	358.04	463.85	680.53	14.40
Yunnan	327.78	430.74	522.67	596.06	874.90	903.41	1534.95	35.76
Tibet	33.72	42.90	52.85	73.12	141.04	157.87	511.51	12.47
Shaanxi	423.57	483.08	551.85	662.77	872.65	899.70	1456.75	30.90
Gansu	373.31	366.07	406.45	486.80	602.68	720.86	912.32	18.49
Qinghai	111.65	110.20	129.43	147.13	178.78	193.72	204.66	3.69
Ningxia	102.24	134.73	136.67	206.05	301.91	349.88	606.35	15.31
Xinjiang	902.79	897.63	1029.57	1176.10	1735.08	2150.18	2477.16	58.56
Total	25,799.36	32,817.17	33,828.22	42,868.14	64,035.79	69,008.78	81,118.79	1989.90

## Supplementary Materials

The following are available online at https://www.mdpi.com/article/10.3390/ijerph182413031/s1. Table S1. The temporal dynamics of gross product of primary industry in China; Table S2. The temporal dynamics of gross product of secondary industry in China; Table S3. The temporal dynamics of gross product of tertiary industry in China.
