# Supplementary material for: Spatio–Temporal Heterogeneity of Urban Expansion and Population Growth in China
Source: Int J Environ Res Public Health. 2021 Dec 10;18(24):13031. doi: 10.3390/ijerph182413031 (PMC8701062; doi:10.3390/ijerph182413031)
Supplement: Supplementary file 1 [file ijerph-18-13031-s001.zip › ijerph-1494660-supplementary.pdf]

Table S1. The temporal dynamics of gross product of primary industry in China.

Province	Primary Industry (100 Million Yuan)							Change Rate (100 Million Yuan)/yr
	1990	1995	2000	2005	2010	2015	2018	
Beijing	43.70	72.20	79.30	86.90	122.80	140.40	118.70	3.07
Tianjin	27.32	60.80	73.69	112.38	131.71	162.31	172.71	5.21
Hebei	90.22	709.39	937.80	1473.62	2576.39	3446.53	3206.32	123.48
Shanxi	221.06	308.33	328.78	486.44	1012.78	1429.81	849.86	36.82
Inner Mongoria	111.14	240.22	347.23	589.56	1134.63	1682.00	1735.33	63.59
Liaoning	165.19	395.03	497.73	876.30	1667.31	2417.13	2033.30	81.32
Jilin	280.55	535.97	691.72	1174.37	1993.22	2950.91	2175.66	89.42
Heilongjiang	118.24	318.84	589.93	942.01	1768.58	3027.22	2909.48	111.58
Shanghai	34.24	59.82	76.68	90.26	117.79	125.53	104.37	2.92
Jiangsu	491.48	990.69	984.39	1362.66	2386.05	3550.76	4128.52	128.81
Zhejiang	224.60	573.52	687.10	887.13	1360.20	1820.44	1909.92	61.28
Anhui	356.81	937.66	651.31	918.50	1284.38	1582.85	2602.44	62.91
Fujian	123.66	446.65	640.58	856.78	1373.82	2118.09	2379.82	80.40
Jiangxi	174.11	383.06	456.28	692.25	1161.24	1691.02	2105.36	67.24
Shandong	1256.28	1087.64	1420.03	2034.19	3349.37	4542.78	4948.87	147.67
Henan	340.07	788.74	1145.92	1892.35	3187.81	4227.32	4209.32	153.44
Hubei	261.94	783.74	843.09	1070.76	2013.05	3358.50	3547.52	119.95
Hunan	236.38	666.25	876.88	1248.76	2113.15	3287.25	3153.40	113.69
Guangdong	390.07	934.97	1274.07	1518.30	2308.50	3386.82	3831.45	120.52
Guangxi	201.84	325.44	599.82	961.81	1663.22	2577.75	3000.92	103.34
Hainan	45.71	128.90	192.00	295.86	526.89	854.72	1000.11	34.38
Chongqing	100.40	264.19	284.87	463.40	659.10	1067.72	1378.27	42.78
Sichuan	486.05	944.25	952.24	1483.42	2470.88	3675.28	4208.23	134.25
Guizhou	261.93	270.10	389.66	441.84	676.62	1704.31	2159.63	64.67
Yunnan	222.14	310.04	425.46	664.52	1170.17	2130.64	2483.60	82.37
Tibet	14.10	23.48	36.39	48.04	68.72	98.04	130.25	3.90
Shaanxi	68.59	172.00	267.67	431.96	989.53	1584.70	1821.09	65.13
Gansu	120.73	128.86	196.93	312.51	556.05	895.17	875.04	30.94
Qinghai	53.52	55.54	60.12	63.52	134.92	216.80	264.95	7.57
Ningxia	16.59	9.97	13.10	24.58	53.48	237.78	280.84	9.42
Xinjiang	94.62	240.71	288.18	510.00	1038.43	1409.66	1692.09	58.32
Total	8703.80	15379.22	18560.74	26481.95	44050.49	64726.87	68997.22	2265.79

Table S2. The temporal dynamics of gross product of secondary industry in China.

Province	Secondary Industry (100 Million Yuan)							Change Rate (100 Million Yuan)/yr
	1990	1995	2000	2005	2010	2015	2018	
Beijing	262.00	643.60	1040.60	2045.60	3387.90	4660.60	5647.70	196.53
Tianjin	181.38	518.55	863.83	2165.83	4937.50	7918.10	7609.81	306.92
Hebei	244.72	1226.05	2554.94	5297.70	10736.21	14533.60	14849.08	586.66
Shanxi	952.19	2021.17	2749.45	7137.96	15588.63	19458.69	7519.73	525.13
Inner Mongoria	100.09	292.59	576.63	1786.82	6852.27	9852.90	6571.61	339.35
Liaoning	540.84	1488.65	2256.12	4248.66	10933.36	13051.47	10510.40	465.99
Jilin	442.51	795.47	1373.64	2963.12	8397.71	13085.27	11884.96	492.69
Heilongjiang	321.48	789.46	1767.97	2481.46	5560.51	5694.49	5133.11	211.58
Shanghai	505.60	1430.47	2231.93	4451.11	7376.81	8259.03	9732.54	346.05
Jiangsu	954.58	2970.05	4133.31	10400.48	22117.60	33648.01	42670.35	1508.32
Zhejiang	408.49	2057.58	3716.80	7237.49	14097.97	19759.59	31278.39	1013.11
Anhui	459.59	1275.44	1057.12	2166.58	1836.39	6461.96	14304.85	377.21
Fujian	160.75	931.23	1924.64	3194.90	7330.35	13060.97	17267.12	595.13
Jiangxi	127.89	433.62	736.61	1858.61	5107.82	8775.89	11316.37	401.87
Shandong	2375.97	2441.92	4223.86	10971.97	21945.15	30446.04	35730.54	1287.48
Henan	394.67	1443.29	2340.23	5578.50	13349.39	18503.69	22479.88	824.15
Hubei	370.02	1178.44	1924.32	2777.50	7533.52	15351.82	19208.03	664.79
Hunan	266.97	805.56	1352.81	2649.84	8113.10	14589.47	14999.84	582.59
Guangdong	594.77	2746.28	4937.00	11271.71	23096.53	34471.63	41526.27	1512.01
Guangxi	210.62	433.75	604.26	1536.07	4504.53	7732.44	8100.28	312.76
Hainan	20.19	78.48	103.97	240.83	546.05	875.82	1095.79	38.81
Chongqing	135.62	492.67	760.03	1577.66	3574.06	7195.00	8328.79	300.95
Sichuan	443.46	1059.05	1675.01	3172.49	8854.04	16361.53	18606.71	681.49
Guizhou	293.32	319.50	522.65	832.60	1909.15	4522.35	5876.85	194.33
Yunnan	244.73	545.88	857.29	1495.10	3387.87	5574.58	7104.72	244.19
Tibet	3.57	13.24	27.05	63.52	163.92	376.28	628.37	19.88
Shaanxi	144.35	363.91	757.80	1872.42	5394.99	8795.62	11710.80	413.22
Gansu	183.71	269.13	424.99	855.31	1964.31	2488.94	2788.61	102.51
Qinghai	172.52	191.30	202.09	270.37	768.87	1208.99	1235.19	42.95
Ningxia	24.88	44.47	73.21	172.37	420.74	1514.35	1796.43	63.06
Xinjiang	83.50	283.97	537.58	1164.80	2592.15	3616.94	4922.97	171.15
Total	13694.91	31850.51	50818.25	107045.67	236817.64	357101.72	408748.70	14975.11

Table S3. The temporal dynamics of gross product of tertiary industry in China.

Province	Tertiary Industry (100 Million Yuan)							Change Rate (100 Million Yuan)/yr
	1990	1995	2000	2005	2010	2015	2018	
Beijing	195.10	791.90	2092.90	5008.90	10930.90	18884.70	24553.60	869.15
Tianjin	102.25	352.62	764.36	1669.73	4274.56	8714.26	11027.12	389.59
Hebei	134.84	878.50	1861.06	3492.39	7303.89	12460.89	16832.72	578.33
Shanxi	321.68	783.96	1372.21	3053.83	7633.18	13275.33	8390.39	420.28
Inner Mongoria	71.40	236.42	493.83	1738.73	4899.31	9024.42	8577.86	353.58
Liaoning	260.23	1111.66	1892.43	3640.65	8050.15	13206.43	13189.13	514.60
Jilin	290.93	610.08	1226.34	2583.14	5937.78	10382.45	13680.95	473.54
Heilongjiang	151.26	498.28	1156.40	1977.70	4066.27	6932.98	8563.83	302.49
Shanghai	241.82	1027.79	2503.54	4824.17	9942.25	17274.62	22842.96	792.24
Jiangsu	490.17	1780.01	3050.86	13556.33	39472.40	34737.46	47379.70	1803.46
Zhejiang	205.15	1135.02	2315.39	5345.62	11400.88	21458.35	29950.83	1018.70
Anhui	303.90	942.58	918.53	2001.28	1965.59	3986.91	13717.11	336.09
Fujian	114.58	746.38	1584.02	2516.03	5758.57	10744.03	16141.63	527.98
Jiangxi	99.99	344.56	675.31	1433.42	3162.43	6385.62	8581.56	293.45
Shandong	1474.99	1577.68	2938.61	6109.72	14889.70	28083.70	37192.19	1262.85
Henan	239.44	853.89	1600.70	3152.70	6703.68	14522.21	21678.41	706.15
Hubei	264.20	842.04	1558.39	2712.77	6243.85	12348.21	18104.54	593.55
Hunan	166.79	703.68	1416.14	2706.21	6129.43	12550.16	19470.56	629.40
Guangdong	494.13	2209.77	4385.57	10182.58	22308.30	40826.06	55689.20	1913.51
Guangxi	213.47	425.60	727.92	1494.69	3395.25	6495.57	9276.81	307.30
Hainan	36.52	155.87	230.85	382.06	991.56	1972.22	2736.15	91.46
Chongqing	91.73	366.20	746.10	1445.16	3724.33	7527.08	10656.13	358.50
Sichuan	326.73	872.94	1512.67	2854.74	6118.18	12008.04	19089.20	605.91
Guizhou	279.00	329.85	462.21	764.00	2034.45	8020.88	12053.36	379.41
Yunnan	181.06	370.99	725.77	1333.00	2845.40	6160.36	8173.45	276.15
Tibet	10.03	19.39	54.37	137.24	274.82	553.11	719.01	25.09
Shaanxi	114.34	334.23	736.54	1576.76	3685.84	7222.33	10393.20	347.98
Gansu	148.71	208.98	424.99	769.50	1521.79	3364.13	4520.53	150.29
Qinghai	97.37	97.37	132.35	206.22	483.68	934.10	1288.09	40.93
Ningxia	17.34	34.71	60.64	184.56	461.45	1164.13	1670.85	55.62
Xinjiang	95.89	290.17	537.80	929.34	1766.69	4208.97	5584.02	187.00
Total	9310.90	23198.89	42642.22	92580.28	211878.42	361100.58	488608.10	16767.48
